# The Value of Troponin as a Biomarker of Chemotherapy-Induced Cardiotoxicity

**DOI:** 10.3390/life12081183

**Published:** 2022-08-03

**Authors:** Victorita Sorodoc, Oana Sirbu, Catalina Lionte, Raluca Ecaterina Haliga, Alexandra Stoica, Alexandr Ceasovschih, Ovidiu Rusalim Petris, Mihai Constantin, Irina Iuliana Costache, Antoniu Octavian Petris, Paula Cristina Morariu, Laurentiu Sorodoc

**Affiliations:** 1 Department of Internal Medicine, Clinical Emergency Hospital Sfântul Spiridon, 700111 Iasi, Romania; victorita.sorodoc@umfiasi.ro (V.S.); raluca.haliga@umfiasi.ro (R.E.H.); alexandra.rotariu@umfiasi.ro (A.S.); alexandr.ceasovschih@umfiasi.ro (A.C.); ovidiu.petris@umfiasi.ro (O.R.P.); mihai.s.constantin@umfiasi.ro (M.C.); paulacristina571@yahoo.com (P.C.M.); laurentiu.sorodoc@umfiasi.ro (L.S.); 2 Faculty of Medicine, University of Medicine and Pharmacy Grigore T. Popa, 16 Universitatii Street, 700115 Iasi, Romania; irina.costache@umfiasi.ro (I.I.C.); antoniu.petris@umfiasi.ro (A.O.P.); 3 Department of Cardiology, Clinical Emergency Hospital Sfântul Spiridon, 700111 Iasi, Romania

**Keywords:** cardiotoxicity, troponin, biomarkers, cancer, chemotherapy, cardiac dysfunction, heart failure

## Abstract

In cancer survivors, cardiac dysfunction is the main cause of mortality. Cardiotoxicity represents a decline in cardiac function associated with cancer therapy, and the risk factors include smoking, dyslipidemia, an age of over 60 years, obesity, and a history of coronary artery disease, diabetes, atrial fibrillation, or heart failure. Troponin is a biomarker that is widely used in the detection of acute coronary syndromes. It has a high specificity, although it is not exclusively associated with myocardial ischemia. The aim of this paper is to summarize published studies and to establish the role of troponin assays in the diagnosis of cardiotoxicity associated with various chemotherapeutic agents. Troponin has been shown to be a significant biomarker in the diagnosis of the cardiac dysfunction associated with several types of chemotherapeutic drugs: anthracyclines, anti-human epidermal growth factor receptor 2 treatment, and anti-vascular endothelial growth factor therapy. Based on the data available at this moment, troponin is useful for baseline risk assessment, the diagnosis of cardiotoxicity, and as a guide for the initiation of cardioprotective treatment. There are currently clear regulations regarding the timing of troponin surveillance depending on the patient’s risk of cardiotoxicity and the type of medication administered, but data on the cut-off values of this biomarker are still under investigation.

## 1. Introduction

Cancer, together with heart disease, is the leading cause of death in developed countries. Advances in cancer treatment have led to significant improvements in the survival of these patients. The cardiotoxic side effects of cancer therapy have contributed to increased mortality and morbidity during and after the cancer treatment [1]. Cardiovascular mortality is up to 10-fold higher in cancer survivors than in the general population, pointing to the importance of conducting a cardiovascular toxicity risk assessment, including the early identification of the cardiac effects of cancer therapies [2].

Cardiac dysfunction associated with cancer treatment is the main cause of mortality in cancer survivors. The mortality rate is recorded to be up to 60% in the first 2 years after therapy [3]. The most commonly associated drugs with cardiotoxicity are anthracycline (AC) and monoclonal antibodies (such as trastuzumab). Other new agents, such as vascular endothelial growth factor (VEGF) inhibitors, immunotherapies, and proteasome inhibitors, can also cause cardiac dysfunction [4].

Evidence from past studies has confirmed the possibility of preventing cardiac dysfunction by promptly initiating cardio-protection with angiotensin-converting-enzyme inhibitors (ACEI) or beta blockers. Therefore, the early detection of cardiotoxicity with precise diagnostic methods and the prediction of patients that would benefit from cardioprotective treatments is essential [5].

## 2. Cardiotoxicity

Cancer-treatment-related cardiac dysfunction can be caused by chemotherapy, immunotherapy, and molecular targeted therapy. The cardiovascular complications associated with cancer treatments include heart failure (HF), acute and chronic coronary syndromes, arrythmias, hypertension, venous thromboembolism, and pericardial disease [3]. Cardiotoxicity represents a decline in cardiac function, with different criteria for definition over the years, and the purpose of this paper is to assess the role of troponin in the diagnosis of cardiotoxicity.

The risk factors associated with an increased risk of cardiotoxicity include an age of over 60 years old, smoking, dyslipidemia, obesity, and a history of diabetes, coronary artery disease, atrial fibrillation, or HF. Patients with any of these risk factors, together with the potential cardiotoxic effects of specific types of chemotherapy, are at a high risk of experiencing cardiac dysfunction associated with cancer treatment [6].

The diagnosis of cardiotoxicity associated with chemotherapy has evolved over the years, from endomyocardial biopsies to the imagistic detection of a decline in cardiac function, and recently, by using the association of cardiac biomarkers (NT-proBNP and troponin) that have been studied during the last years (8). The ejection fraction was proposed as the main tool for the diagnosis of cardiotoxicity by the Task Force for Cancer Treatments of the European Society of Cardiology, published in 2016. The cardiac dysfunction associated with treatments for cancer was defined as a reduction in the left ventricular ejection fraction (LVEF) to less than 50%, or a reduction in the baseline by more than 10% coupled with an LVEF of less than 55%. Global longitudinal strain, as an early sign of cardiotoxicity, may be used depending on the availability [7].

A more recent document (2022) from the International Cardio-Oncology Society divided cardiac dysfunction into asymptomatic and symptomatic groups and delivered a classification system of three grades for cardiac dysfunction in asymptomatic patients, depending on the severity of the LVEF detected by echocardiography. For all the stages, biomarkers and measurements of the GLS (global longitudinal strain) could be used in addition to assess the severity of the disfunction:

- Mild asymptomatic cardiac dysfunction consists of an LVEF > 50% with a rise in troponin (cTn) or natriuretic peptides and a reduction of >15% in the GLS from baseline;
- Moderate asymptomatic cardiac dysfunction is defined by a reduction in the LVEF of ≥10%, which would thus be in the 40–49% range, or a smaller change in the LVEF associated with a significant fall in the GLS and/or a rise in cardiac biomarkers;
- Severe asymptomatic cardiac dysfunction is defined by a reduction in the LVEF to <40% and is associated with a poor prognosis [8].

Symptomatic cardiac dysfunction associated with cancer therapy is associated with symptoms and signs of volume overload and/or inadequate perfusion caused by functional and structural changes to the heart, with a decrease in LVEF and increased biomarkers [8].

Anthracycline (AC), a class of chemotherapeutic agents, induces irreversible cardiac dysfunction, leading to severe heart failure and death in a dose-dependent manner. The changes occur predominantly in the first year following the completion of treatment, although there are cases of cardiotoxicity that appear years after the exposure. Trastuzumab toxicity is not associated with structural abnormalities, is independent of the dosage, and can be partially or completely reversed with early therapeutic interventions, such as the cessation of the causative drug or cardioprotective treatment with ACEI and beta blockers. These side effects can lead to suboptimal cancer treatment regimens and increased mortality and morbidity [9]. 

Different mechanisms are involved in the decline of cardiac function due to cancer therapies. The cardiomyocyte damage can be:

- Direct or endogenous (e.g., AC)—also called primary cardiomyopathy—which is a consequence of the direct toxic effects of chemotherapy on the myocardium and is caused by myocardial cell loss, necrosis, and apoptosis mediated by oxidative stress [10].
- Indirect (e.g., trastuzumab, VEGF inhibitors), which is caused by factors that do not have a direct toxic effect on cardiomyocytes, but that contribute to a decline in cardiac function. Indirect cardiomyopathy is determined by alterations in the perfusion, innervation, or hormonal background (vasoconstriction, vasospasm). Trastuzumab causes functional abnormalities, though in patients with previous AC treatment, it may exacerbate injury and myocyte death by inhibiting anti-apoptotic pathways [11].
- Caused by inflammatory cell infiltration in the myocardium, which can lead to myocarditis (immune checkpoint inhibitors—ICS) [11].

Cardiomyopathies caused by cancer therapy are rarely a consequence of a single mechanism, so this classification can be used to foster the proper selection of the care and produce the best outcomes [12]. The latest studies on the incidence and reversibility of cardiotoxicity have led to a withdrawal of the concept of type I and II cardiotoxicity, and have brought to our attention the Royal Brompton Hospital classification of myocardial toxicity [13]. This classification is more applicable to clinical practice, and includes early abnormalities that could predispose a patient to future susceptibility and identify high-risk patients that could benefit from preventive treatment. This classification includes six stages:

1. Early biochemical cardiotoxicity, which is represented by an increase in cardiac biomarkers (troponin or brain natriuretic peptide—BNP) with normal cardiac imaging.
2. Early functional cardiotoxicity, which is characterized by grade III–IV diastolic dysfunction or a reduction in the GLS and normal biomarkers.
3. Early mixed cardiotoxicity, which involves the presence of a normal LVEF with increased levels of biomarkers and a reduction in the GLS, or diastolic dysfunction.
4. Symptomatic heart failure with preserved EF.
5. Asymptomatic LV systolic dysfunction, which is indicated by a reduction in the LVEF to less than 50%, or a reduction of more than 10% resulting in a total LVEF < 55%.
6. Symptomatic LV systolic dysfunction, which is represented by a symptomatic reduction in the LVEF to <50%, or a reduction of >10% resulting in a total LVEF < 55% [14].

Echocardiographic changes used for the diagnosis of cardiotoxicity have a low diagnostic sensitivity and low predictive power for the diagnosis of subclinical myocardial injury. The lack of standardization in defining cardiac dysfunction across clinical trials and chemotherapeutic agents has led to multiple other questions regarding the role of this diagnostic tool in detecting cardiac dysfunction [15]. Echocardiographic measurements of the LVEF have several limitations, which include intra-observer or inter-observer variability and a lack of sensitivity for detecting early subclinical myocardial changes. Unfortunately, these changes appear late and are often an irreversible effect of the drugs used for chemotherapy [16]. The use of new techniques, such as three-dimensional echocardiography and contrast echocardiography, have resulted in a significant improvement to the accuracy assessment of the LVEF. The GLS can be used to detect early subclinical changes in the systolic function of the left ventricle [16].

Cardiac magnetic resonance imaging (CMR) remains the gold standard for the rigorous quantification of the LVEF, but its use is limited by the cost and the requirement for expert interpretation [4].

As a result of the complexity of the cardiovascular complications of cancer treatment and the extensions of cardiovascular toxicity beyond changes in the LVEF, there is a growing expectation for noninvasive diagnostic tools that can be used for the early detection of patients at a high risk of developing chemotherapy-induced cardiotoxicity. Finding a biomarker able to detect heart injury represents a desirable tool for the initial diagnosis of cardiotoxicity [17].

### 2.1. Biomarkers for Cardiotoxicity

The limitations of the mentioned imagistic method and the need to identify cardiotoxicity in early stages has led to the analysis of sensitive and specific markers of myocardial damage [18]. The most frequently used biomarkers for the diagnosis of cardiotoxicity are troponins and NT-pro BNP. A troponin assay offers a complementary diagnostic tool for patients receiving chemotherapy, and troponin assays represent a good tool for surveillance, prognosis assessment, and providing a guide for selecting and monitoring the treatment response in oncology patients [7,19].

The perfect biomarker should provide rapid and low-cost measurements, offer objectivity, be precise (the normal ranges are well-defined), be widely available, be well-studied, and offer reproductible results. The biomarker should provide better diagnostic confidence as well as information about disease progression, risk stratification, and treatment responses [20]. The detection of a specific biomarker is minimally invasive, has a significantly lower cost than echocardiography or nuclear techniques, and reflects minimal cardiomyocyte damage or small hemodynamic fluctuations. The interpretation of the results is not dependent on the expertise of the doctor, thus avoiding inter-observer variability [21]. Biomarkers have an important role in the initial evaluation and management of patients who are known to have or are at risk for cardiovascular disease. These blood tests can be good tools for identifying patients with a high risk of adverse cardiovascular effects before the initiation of therapy, and are a good addition to imaging in the detection of subclinical diseases during active therapy. For the future, an idealistic role for biomarkers would be the tailoring of oncologic regimens or the initiation of preventative cardiovascular treatments based on cardiac biomarker profiles [22]. 

Most of the published studies have identified the biomarkers for cardiovascular disease in patients receiving cancer treatment. However, the specificity of these biomarkers in various categories of patients is difficult to assess due to the comorbid conditions associated with cancer pathology, such as infections, hypotension, renal dysfunction, and other systemic illnesses. All these known conditions can lead to increased biomarkers in the absence of cardiac dysfunction [4].

### 2.2. Troponins

Troponins are biomarkers used for the diagnosis of acute coronary syndromes. Cardiac troponins (cTnI and cTnT) are largely used to detect cardiac toxicity, as they have a high specificity for cardiac injury and cardiomyocyte necrosis. The highly sensitive assays allow for the possibility of detecting small amounts of myocyte damage, enabling the application of therapeutic interventions to minimize cardiotoxicity before the development of irreversible left ventricular (LV) dysfunction. The persistent elevation of cTn I has the role of detecting a higher degree of LV dysfunction and a higher percentage of cardiac events than the transient elevation of cTn levels [11]. 

Troponins are a complex of regulatory proteins that are part of the skeletal and cardiac muscle, but not smooth muscle. Their function consists of regulating muscle contractions. Sarcomeres represent the fundamental units of a myofibril, and they are formed from seven actin monomers, a strand of tropomyosin, and a troponin complex [23]. There are three subunits of troponins: troponin C, cTnT, and cTnI. cTnT and cTnI have two specific isoforms that are distinct for cardiac and skeletal muscles. The two isoforms of troponin C are not different in cardiac and skeletal muscles. Each subunit of troponin has a specific function: cTnI is bound by actin and keeps the troponin–tropomyosin complex steady, cTnT anchors the components of the tropomyosin to the actin thin filament, and troponin C contains the binding sites for calcium, which help initiate contractions [24]. Diastolic relaxation occurs when cTnI binds to actin and its access to the myosin binding site is impeded, maintaining tropomyosin stability. A systolic contraction starts when a calcium influx causes the release of cTnI from actin, liberating its myosin binding site and facilitating force development [25]. 

High-sensitivity cardiac troponin (hs-cTn) is more specific, being able to detect levels 10- to 100-fold lower than the original technique and improving the accuracy of myocardial injury diagnoses. When hs-cTnT and hs-cTnI are compared, the latter is superior in the early detection of lesions, and its levels are not affected by the circadian rhythm [9]. There is no evidence that cTnT can be released by noncardiac tissues, but in patients with injured skeletal muscle, some proteins can be detected by cTnT assays, making the cTnT a less-specific marker of cardiac injuries [5].

The troponins cTnI and cTnT are expressed exclusively in the myocardium, and their release into circulation is an effective indicator for evaluating myocadic integrity. After cardiomyocyte necrosis, cTn is released, which has a high sensitivity and specificity for the diagnosis of myocardial infarctions. A high level of cTn can be detected before echocardiographic changes; therefore, it can become an important assessment tool for detecting subclinical cardiac dysfunction. The cTn measurement has a high specificity for the heart, but is not specific for disease [9]. Myocardial injury can be determined by reversible or irreversible impairment. If there is irreversible damage, the release of proteins from the myocardium is caused by the apoptosis or necrosis of myocardial cells [26]. If the disturbance is reversible, the release of cTn is secondary to an increased cell wall permeability or the formation and release of membrane cysts. The other mechanisms of troponin release are myocardial cell turnover and the release of cell breakdown products [5]. 

Changes in hs-cTn concentrations during short periods of time can discern between acute disease, when there is an acute injury of the cardiomyocyte with rapid increases and decreases in the cTn, and chronic cardiomyocyte injury, with a persistent slight elevation of hs-cTn [27]. 

A small concentration of cTn can be detected even in normal subjects, and the diagnosis of myocardial damage is considered pronounced if the levels of cTn are higher than the 99th percentile of the upper reference limit. High levels of cTn can be found, not only in myocardial injuries, but also in well-known inflammatory or mechanical conditions [5].

### 2.3. Mechanism of Troponin Release

**Figure 1:**
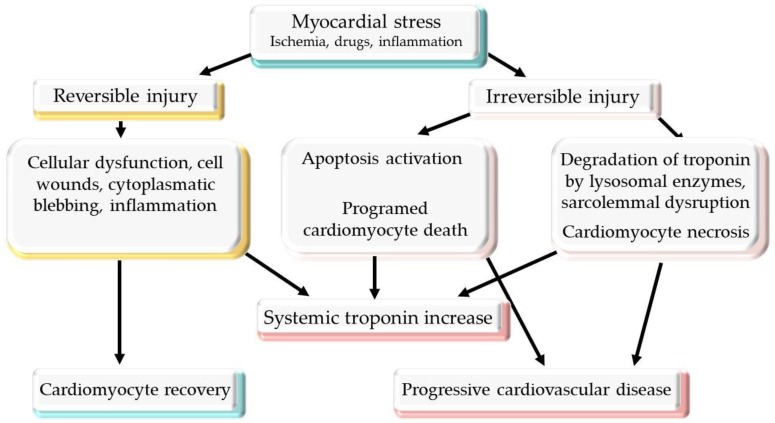
Potential mechanisms of troponin release from injured myocardium.

The vast majority of cTn is stationary in the sarcomere, and a small fraction exists as a soluble pool. In patients with cardiac ischemia, the first peak of cTn release originates from the bound cTn pool. After that, the degradation of the contractile apparatus is responsible for most of the cTn release [28]. Until recently, it was believed that cardiomyocytes cannot be repaired or replenished; however, the newest evidence supports the renewal of cardiomyocytes by mitosis and cellular restoration from stem cells. The renewal of cardiomyocytes could be associated with the release of cTn into the bloodstream. cTn can be released from the myocardium in the absence of necrosis, during physiological situations (athletes after endurance exercise) or after atrial pacing or dobutamine stress test. This means that there are other mechanisms by which cTn is released from the myocardium [29]. One of the possible mechanisms is programed cellular death, which can appear in the absence of ischemia and can lead to HF. In response to myocardial injury, cTn could be released from an enhanced apoptosis rate [30]. The possibilities for the release of cTn into the bloodstream are represented in Figure 1. 

Different cell wounds can lead to cardiomyocyte membrane injury. The cells have the ability to repair lesions larger than 10 μm^2^ within seconds. During this period of time, some proteins could be released into the bloodstream, including cTn, which could possibly explain the high levels of cTn in the absence of myocardial necrosis. The extent of the cTn elevations explained by cell wounds that we find in clinical settings remain to be shown [30].

Another theory involves the release of cTn after bleb formation. These are buds of membrane cells that are formed after transient ischemia and contain cTn, which is released into circulation or captured into the cell after the reestablishment of the oxygen supply. Oxidative stress, reactive oxygen species, neurohormonal activation, inflammatory cytokines, acid–base disturbances, or neurohormonal activation can lead to the release of cTn into the bloodstream [23,31,32]. The understanding of different mechanisms on troponin release from a damaged myocardium could help make the distinction between high troponin levels associated with necrosis and other possible reversible causes of troponin release, which are associated with mechanisms encountered in cardiotoxicity.

The intra-individual variability of two consecutive measurements of hs-cTn is low, so a difference in the levels of the same hs-cTn subtype in a single patient at two different moments should be considered significant if it is higher than 30% (approximately 3–5 ng/L). An increase of 3–5 ng/L in the cTn concentrations between two measurements is correlated with the necrosis of about 10–20 mg of myocardial tissue, which is undetectable even with the most sensitive cardiac imaging techniques [33]. This evidence suggests that, when using hs-cTn as a biomarker for cardiotoxicity detection, the measurement should be performed at baseline, prior to the cardiotoxic treatment, and the monitoring of the biomarker changes during the treatment should be performed using the same subtype of hs-cTn and at the same laboratory, if possible [5].

We will discuss the classes of drugs that could lead to increased levels of cTn and present the existing evidence regarding the role of cTn in the detection of cardiotoxicity and the specific indications for short- and long-term monitoring.

### 2.4. Anthracycline

Anthracyclines (ACs) are chemical compounds whose action is to kill tumor cells by interrupting their mitotic activity and their high metabolic demand, inhibiting DNA or RNA synthesis. Other effects of AC therapy include the inhibition of topoisomerase IIa, an enzyme involved in DNA transcription and replication, and the induction of oxidative stress, which damages DNA, lipids, and proteins by iron mediation and histone modification [12]. 

The incidence of cardiac dysfunction associated with AC in a study of 2625 patients followed for 5.2 years was 9%, and the majority of cases occurred within 1 year of treatment [9]. The incidence was related to the cumulative dose. If the dose of Adriamycin was 400 mg/m^2^, the incidence was 5%; at a dose of 550 mg/m^2^, the proportion of HF was 26%; and the incidence increased to 48% when the dosage was 700 mg/m^2^ [34]. The factors associated with cardiovascular toxicity are female sex, cumulative doses, intravenous bolus administration, and a history of hypertension, cardiovascular diseases, valvular heart disease, or diabetes mellitus [1].

There are three types of cardiac dysfunction associated with AC treatment:

- Acute toxicity in less than 1% of patients, which may occur after the first cycle of treatment or the first dose and is more common in the elderly, probably owing to underlying cardiac diseases. The forms of manifestation are represented by arrythmias, pericarditis, myocarditis, and acute LV dysfunction and are usually transient [35].
- Early-onset chronic cardiotoxicity, which is more common (20–30% with asymptomatic decreases in the LVEF, or symptomatic HF in 1.6–2.1%) and can lead to irreversible cardiac dysfunction [35].
- Late or chronic cardiomyopathy related to cumulative doses of AC, with an onset after more than 1 year and which is expressed as an arrythmia or LV dysfunction [10].

It has been shown that patients receiving chemotherapy that had an elevation of cTnI levels developed LV dysfunction in the following seven months, while in patients with negative cTnI levels, the reduced LVEF was smaller and transient (less than three months). The authors concluded that cTnI is a sensitive and reliable marker for the early detection of minor cardiac dysfunction [36]. Studies that have evaluated the role of cTn in the cardiotoxicity associated with AC are summarized in Table 1. In order to assess whether high levels of hs-cTnI are associated with a long-term reduction in the LVEF, a study was conducted that evaluated 703 patients treated with high doses of chemotherapy for 3 years. In patients without increased levels of hs-cTnI, the LVEF remained at the same levels and a small number of cardiac events occurred (1%). Patients with high levels of hs-cTnI after the chemotherapy had a higher incidence of cardiac events (0.4%—sudden death; 0.3%—cardiac death; 5%—asymptomatic LV dysfunction; 7%—HF; 0.4%—acute pulmonary edema; 2%—life-threatening arrythmia; 0.3%—conduction disturbances). In these patients, careful monitoring is essential and prophylactic strategies to prevent cardiotoxicity should be implemented. The study showed that hs-cTnI has a high negative predictive value of 99% for patients with no elevation of hs-cTnI, and a positive predictive value of 84% for future cardiac events in patients with elevated hs-cTnI levels [37]. These studies suggest that measurements of hs-cTnI prior to and during therapy could identify patients at a high risk for cardiotoxicity.

A recent study that evaluated 80 patients with breast cancer treated with AC showed that the value of hs-cTnT increased gradually during AC therapy, and that it reached a maximum peak after 1 month, with similar patterns observed in patients with and without cardiotoxicity. In addition, a linear dependence of cTnT with age was observed, but it was not correlated with the level of toxicity [38].

A meta-analysis of 61 studies with 5691 patients treated with AC, AC followed by human epidermal growth factor receptor 2 (HER2) inhibitors, or HER2 inhibitors has shown that, in patients with elevated levels of cTn, the likelihood of LVEF dysfunction was higher than in patients with normal cTn levels (OR: 11.9, 95% CI: 4.4–32.1). This association was most pronounced under high-dose regimens of chemotherapy, and the specificity and sensitivity values of cTn for diagnosis were 69% and 87%, respectively. For the prediction of LV dysfunction, the negative predictive value was 93%, showing a potential benefit of cTn as a screening tool for LV dysfunction associated with cancer therapy [39]. 

**Table 1:** The role of troponin in cardiotoxicity associated with anthracycline treatment.

Study	Patients	Design	Type of Cancer	Chemotherapy	Type of cTn	Determination of cTn	Outcomes
Cardinale et al., 2000 [36]	204	PR	Breast cancer Ovarian carcinoma Small cell lung cancer Non-Hodgkin’s lymphoma Hodgkin’s disease	AC, radiotherapy	cTnI	Before, immediately after, and then at 12, 24, 36, and 72 h after every single cycle of high-dosage chemotherapy	LVEF decreased in cTnI group (cTnI > 0.5 ng/mL)
Auner et al., 2003 [40]	78	PR	Acute lymphoblastic leukemia Acute myeloid leukemia Non-Hodgkin’s lymphoma	AC	cTnT	Baseline and after every cycle	Elevated cTnT (>0.03 ng/mL) was associated with a significantly greater decrease in LVEF
Cardinale et al., 2004 [37]	703	PR	Breast cancer Ewing’s sarcoma Hodgkin’s disease Myeloma Non-Hodgkin’s lymphoma Ovarian carcinoma Small-cell lung cancer	AC, radiotherapy	cTnI	Baseline, after chemotherapy, at 12, 24, 36, and 72 h after every single cycle, and at 1 month after the last cycle	Patients with high levels of cTnI (>0.08 ng/mL) had a higher risk of cardiac events
Kilickap et al., 2005 [41]	41	PR	Lymphoma Breast cancer Malignant mesenchymal tumor Leukemia Nasopharyngeal carcinoma Thymic carcinoma Neuroectodermal tumor Hepatocellular carcinoma Metastasis of unknown origin Multiple myeloma	AC	cTnT	Baseline, on the 3rd and 5th days following the first dose of anthracycline, and after the last cycle of chemotherapy	High levels of cTn were associated with diastolic dysfunction (decreased E/A ratio, IRT prolongation)
Nistico et al., 2007 [42]	20	PR	Breast cancer	AC, taxanes	cTnT	Baseline, pre- and post-chemotherapy, and 12 months after the end of treatment	No cTnT serum elevations were found
Horacek et al., 2008 [43]	23	PR	Leukemia	AC	cTnT cTnI	Baseline, after the first cycle, after the last cycle, and at 6 months after completion of treatment	cTnI seemed to be superior to cTnT for the early detection of cardiac injury
Feola et al., 2011 [44]	53	PR	Breast cancer	AC	cTnI	Baseline, at 1 month, at 1 year, and at 2 years after the end of the chemotherapy	cTnI elevations were not correlated with changes in LVEF
Morris et al., 2011 [45]	95	PR	Breast cancer	AC, taxanes, trastuzumab	cTnI	Baseline, every 2 weeks during chemotherapy, and at 6, 9, and 18 months	cTnI levels did not correlate with decreased LVEF
Sawaya et al., 2012 [46]	81	PR	Breast cancer	AC, taxanes, trastuzumab	usTnI	Baseline, after AC treatment, and every 3 months until 12 months	Elevated usTnI (≥30 pg/mL) at the completion of the AC treatment was predictive of cardiotoxicity
Onitilo et al., 2012 [47]	54	PR	Breast cancer	AC, trastuzumab	cTnI	Baseline and every 3 weeks until 1 year	A decrease in LVEF was not associated with the levels of cTnI
Blaes et al., 2015 [48]	18	PR	Breast cancer Non-Hodgkin’s lymphoma	AC	cTnT, cTnI, hs-cTnT	Baseline and at 4 weeks after completion of treatment	A decline in LVEF was associated with baseline high hscTnT levels
Malik et al., 2016 [49]	33	PR	Breast cancer	AC	cTnT	Baseline, after every cycle, and at 6 months after the end of the treatment	A decrease in the left ventricular diastolic diameter was associated with high cTnT levels
Jones et al., 2017 [19]	84	PR	Breast cancer Lymphoma Leukemia Leiomyosarcoma	AC	hs-cTnI	Baseline and after every cycle	Smaller AC doses per cycle resulted in less acute cardiomyocyte injury as indicated by hs-cTnI release
Ferreira de Souza et al., 2018 [50]	27	PR	Breast cancer	AC	cTnT	Baseline, after every cycle, and at 3 and 6 months after treatment	A reduction in LVEF and LV mass was more pronounced in patients with cTnT > 10 pg/mL
Demisei et al., 2020 [51]	323	PR	Breast cancer	AC ± trastuzumab	hs-cTnT	Baseline, at 1 month, and after the end of AC treatment at 2 and 4 months	Elevated hs-cTnT (>14 ng/L) was associated with a double risk of cardiotoxicity
Michel et al., 2020 [39]	61 research articles and 5691 patients	Meta-analysis	Various	AC, various high doses of chemotherapy	cTnI, cTnT	Various	In patients with elevated levels of cTn, the likelihood of LVEF dysfunction was higher than in patients with normal cTn
Diaz-Anton et al., 2022 [38]	72	PR	Breast cancer	AC ± trastuzumab	hs-cTnT	Before and after each cycle and at 1, 3, 6, and 12 months after completion of treatment	hs-cTnT increased gradually, reaching a maximum peak at 1 month after the completion of anthracycline treatment

AC—anthracycline, cTn—troponin, hs-cTn—high-sensitivity troponin, LV—left ventricle, LVEF—left ventricular ejection fraction, PR—prospective, us-cTnI—ultrasensitive troponin I.

A study on 323 patients treated with doxorubicin showed that an increased level of hs-cTnT over 14 ng/L after AC treatment increased the risk of cardiac dysfunction by 2-fold (hazard ratio: 2.01; 95% CI: 1.00–4.06, *p* = 0.052). The sensitivity and specificity of hs-cTnT were 60.3% and 62.5%, respectively, for the prediction of cardiac dysfunction within 1 year after the finalization of the doxorubicin treatment. In contrast, patients with levels of hs-cTnT < 5 ng/L at the end of AC treatment had a sensitivity of 100% for cardiac dysfunction at 1 year [51]. 

Another study of 41 patients receiving AC therapy suggested an association between the levels of cTn and the diastolic dysfunction assessed by the E/A ratio and isovolemic relaxation time (IRT) [41].

A small study on 23 patients treated with AC for leukemia suggested that an assessment of cTnI could be superior to cTnT for the early detection of cardiac dysfunction because of the molecular weight and the release kinetics of the two forms of troponin [43].

The goal of a study including 81 patients treated with AC, followed by taxanes and trastuzumab, was to assess the role of blood biomarkers and myocardial strain in cardiotoxicity prediction. Cardiotoxicity was developed by 32% of the patients, and the study showed that us-cTnI (ultra-sensitive cTnI) and the peak systolic longitudinal myocardial strain at the finalization of treatment predicted cardiotoxicity. Concentration levels of us-cTnI ≥ 30 pg/mL after AC treatment were predictive of cardiotoxicity (*p* = 0.04). A combination of us-cTnI and longitudinal strain increased the sensitivity of the cardiotoxicity diagnosis from 74% to 87%, with a negative value of 91% [46].

Not all the studies performed have shown an association between high levels of cTn and cardiac outcomes [44,45,47]. A study on 53 patients with breast cancer treated with AC failed to demonstrate that an elevation of cTn measured 1 month post-treatment could predict cardiac outcomes. The cTn levels were not different between the patients with and without cardiac events [47].

As presented, there are some studies that have highlighted the beneficial role of measuring cTn levels during AC therapy, and others that could not find an association between the levels of cTn and cardiovascular toxicity. Potential explanations for the discrepancies between these studies include a difference in the timing of biomarker measurement, the cutoff values used, and the type of chemotherapy administered [4].

Regardless of the predictive role of cTn in detecting the clinical manifestation of cardiac dysfunction, its increase is an index of subclinical cardiac damage. In this context, some authors have suggested that the administration of ACEI and beta blockers in patients with elevated cTn levels is effective in preventing the development of LV dysfunction [52,53]. Cardinale et al. established that the response to HF treatment (increased LVEF) is determined by the time between the end of chemotherapy and the initiation of HF treatment (beta blockers and ACEI) [54].

The presented studies show that, in patients treated with anthracycline, a positive value of cTn is acceptable, but possibly the best benefit of monitoring cTn in these patients is the ability to exclude subjects that will not develop cardiac dysfunction. Although we do not have specific data about the cut-off values of cTn after chemotherapy, a recent study has evaluated the increase in cTn after radiation treatment and concluded that the cut-off levels for cardiac events were 10 ng/L for hs-cTnT before the initiation of radiation, 16 ng/L for hs-cTnT during treatment, and 12 ng/L for hs-cTnT after radiation [55].

In 2020, the Cardio-Oncology Study Group of the Heart Failure Association and the Cardio-Oncology Council of the European Society of Cardiology published a position statement regarding the type and duration of biomarker monitoring, depending on the cardiotoxicity risk of the patient. Cardiotoxicity risk is evaluated according to the patient’s cardiovascular profile and risk factors relating to preexisting cardiovascular disease and the type or dose of cancer treatment [13].

Patients at low risk are adults treated with low dose of AC, liposomal products or trastuzumab without AC. Patients with moderate risk are more than 50 and less than 64 years old, present 1–2 cardiovascular risk factors (smoking, dyslipidemia, insulin resistance, obesity), are treated with modest dose of AC (doxorubicin 200–400 mg/m^2^, epirubicin 300–600 mg/m^2^), with AC before trastuzumab, or are treated with one of the following classes of drugs: vascular endothelial growth factor (VEGF), tyrosine kinase inhibitors (TKI), Second- and third-generation Bcr-Abl TKI, Proteasome inhibitors, Combination immune checkpoint inhibitors. Patients with high risk of cardiotoxicity are more than 65 years old and presents more than 2 cardiovascular risk factors, cardiovascular disease (severe valvular heart disease, cardiomyopathy, coronary artery disease, heart failure, peripheral artery disease) presented prior cancer therapy and the LVEF is reduced before the initiation of treatment (50–54% or less) and the cTn levels are increased after AC treatment. The patients with high risk are treated with a combination of AC with trastuzumab or VEGF-TKI after AC therapy, high doses of AC (doxorubicin > 400 mg/m^2^, epirubicin > 600 mg/m^2^) and radiation therapy with a modest dose of AC or with a high dose (heart in the radiation field with >30 Gr) [13].

The position statement of the ESC suggests that, in patients with a low or medium cardiotoxicity risk treated with anthracycline, an assessment of cTn should be performed at the baseline, before the fifth cycle, and at 12 months after the end of treatment. For patients with a high cardiovascular risk, the measurement should be made before cycles two, four, and six or before every cycle and at three, six, and twelve months after the final cycle of treatment [13].

### 2.5. Trastuzumab 

Almost 20% of breast cancers express human epidermal growth factor receptor 2 (HER2). An anti-HER2 agent—trastuzumab—was developed and was demonstrated to reduce the disease progression by 40%. HER2 isoforms are also expressed in cardiomyocytes; therefore, trastuzumab treatment is associated with LV dysfunction and HF [56]. This review focused on trastuzumab because is the most studied in the class of HER2 direct therapies. Five approved trastuzumab biosimilars demonstrate similar rates of cardiotoxicity [57].

The studies available on the role of cTn in cardiac dysfunction associated with trastuzumab are presented in Table 2. The exact mechanism of trastuzumab-induced cardiotoxicity is still unknown, but some studies have suggested the following possibilities: the inhibition of cardiomyocyte repair by blocking the HER2 downstream pathway and neuregulin 1, and/or the inhibition of topoisomerase IIb, leading to the increased formation of reactive oxygen species and apoptosis [58].

One of the major side effects of trastuzumab treatment is cardiotoxicity expressed as an asymptomatic decrease in the LVEF and HF. After treatment with trastuzumab alone, the incidence of cardiac dysfunction ranged from 3 to 7% and reached 27% when the treatment was performed in combination with AC [67]. A meta-analysis including 9117 patients across five studies showed that the likelihood of cardiotoxicity after trastuzumab use was 2.45-fold higher than the likelihood without trastuzumab use (95% CI: 1.89–3.16) [68]. 

The clinical outcome of cardiotoxicity associated with trastuzumab treatment is more favorable than that associated with AC, because it is not dependent on the dosage and can be reversed by discontinuing the treatment or by using standard cardiac therapy. Other data have shown that, in patients treated with AC and trastuzumab, the cardiac function does not recover, so it is difficult to separate the cardiotoxicity induced by AC or trastuzumab in patients receiving both chemotherapies [21].

The risk increased in patients that received a combination of chemotherapy, especially including AC; when there was a short duration between the two types of treatment; with increasing age of the patient (>65 years); with chest wall irradiation; for patients with other comorbidities (hypertension, obesity, or diabetes); and for patients with previous cardiac dysfunction [44]. Based on these risk factors, a Canadian study evaluated a score model that predicts the possibility of cardiotoxicity, and established that a low score was associated with a negative predictive probability of 94% for permanent toxicity, and a high score was associated with a positive predictive value of 0.17 [68].

Despite all these data, an elevation of cTn in patients with trastuzumab therapy is usually seen at the shift between the AC and trastuzumab treatments, leading to cardiac injury and dysfunction [12].

One of the largest studies evaluating the role of cTnT and cTnI in predicting the cardiac dysfunction of patients [69] with trastuzumab treatment established that an increased baseline of cTnT and cTnI was associated with a 4-fold increased risk of cardiac dysfunction [62]. A study that included 251 patients with breast cancer receiving trastuzumab treatment, with or without AC in combination, revealed that cardiotoxicity occurred in 17% of the patients and affected mostly the patients with elevated cTnI (62% vs. 5%, *p* < 0.001). The recovery of the LVEF was present in 60% of these patients, and an improvement in the LVEF was less likely in patients with positive cTnI levels during treatment (35% vs. 100%; *p* < 0.001). In a multivariate analysis, the cTnI levels were the only independent predictor for cardiotoxicity related to trastuzumab treatment (HR: 22.9; 95% CI: 11.6–45.5; *p* < 0.001) and of the absence of LV function recovery (HR: 2.88; 95% CI: 1.78–4.65; *p* < 0.001). The patients who received a combination of drugs with AC prior to trastuzumab were more likely to present with positive levels of cTnI, suggesting that the high levels of cTn were determined by the preexisting myocardial damage secondary to the AC treatment [21]. Similar results, confirming that the levels of us-cTnI at the end of AC treatment were a predictor of cardiotoxicity in patients treated with trastuzumab, were demonstrated by a study performed in Tunisia [66]. 

These studies raise some questions regarding the elevation of cTn levels. The most important is related to the relationship between the increased levels of cTn after AC treatment and patients that continue on to receive trastuzumab. If increased levels of cTn occur mostly in patients treated previously with AC and drop over time, regardless of trastuzumab treatment, this could be explained by two mechanisms. First, the mechanism suggests that the increased levels of cTn appear as a consequence of the AC treatment, and are not related to trastuzumab. This possibility is supported by the high levels of cTn detected before trastuzumab treatment and the high levels of cTn that can persist for months after AC treatment. Another possibility is that the increased levels of cTn occur by the direct trastuzumab damage of the already-vulnerable heart after AC administration. Considering the fact that myocardial HER2 expression increases early after AC treatment and disperses in time, this mechanism is also plausible [70]. The studies to come should shed light on this dilemma.

Despite the results presented above, a study on 54 patients receiving adjuvant therapy with trastuzumab did not find an association between the levels of cTn and the risk of cardiac dysfunction [44]. Another study evaluated 95 patients with breast cancer receiving trastuzumab after chemotherapy with AC, and showed that an increase in cTnI at 14 weeks after the beginning of chemotherapy preceded the maximum deterioration of the LVEF, but did not predict or relate to the maximum decline in the LVEF [45]. However, a study aimed at finding a pharmacokinetic–pharmacodynamic (biomarker) model for the prediction of LVEF decreases showed that the maximum concentration of cTnT after AC treatment was an important determinant for reduced LVEF levels resulting from trastuzumab treatment [63].

The studies performed until now have shown conflicting results for the association between cTn and cardiac dysfunction. The most likely explanation for these results is the timing of cTn detection and the previous AC treatment [58]. In this context, major studies to detect a association between the levels of troponin and cardiotoxicity from trastuzumab (with or without AC association) are needed in order to specify the exact timing of cTn detection and its relationship with the presentation of cardiac dysfunction. 

An interesting study showed that cTnI could predict the reversibility of cardiotoxicity induced by trastuzumab. All patients with cardiac dysfunction with cTnI levels < 0.08 ng/mL recovered their normal cardiac function, while only 35% patients with cTnI levels over this threshold recovered [53].

The available recommendations for cTn measurements in patients receiving trastuzumab treatment depend on the cardiotoxicity risk of the patients. For all patients, cTn should be measured before the beginning of the treatment and at 12 months after the completion of therapy. In patients with a low risk, cTn should be measured every four cycles; in medium-risk patients, it should be measured before alternate cycles for 3–6 months and every three cycles for the remaining treatment until 1 year. At 3–6 months after the final cycle, a cTn assay should be performed. Patients with a high risk should have an assessment of cTn before and after every cycle for the first 3–6 months, then every three cycles until 1 year. After the end of the treatment, another measurement should be performed at every 3 months [13].

### 2.6. Immunotherapy

Immune checkpoint inhibitors (ICSs) belong to a class of anticancer treatment that magnifies T cell-mediated immune feedback against cancer cells. The cardiotoxic effects of ICS include myocarditis, cardiomyopathy, arrhythmias, and vasculitis, but myocarditis is the side effect associated with major morbidity and high mortality [71]. 

The incidence of myocarditis associated with ICS treatment was 0.27% in patients receiving a combination of therapies and 0.09% in patients receiving a single ICS. A multicenter registry noted a prevalence of 1.14% for a single treatment, which increased to 2.4% for a combination of therapies [71]. Although myocarditis can appear at any time during ICS treatment, it usually appears early, in most cases occurring in the first 3 months of therapy, and it is associated with a very high mortality rate (38–46%) and a high number of nonfatal major cardiovascular events, such as HF, complete heart block, ventricular arrhythmias, cardiac arrest, or cardiogenic shock [72]. 

According to the recommendations for the diagnosis of myocarditis associated with chemotherapy, the gold standard is a myocardial biopsy. Cardiac magnetic resonance (CMR) is the preferred imagistic modality for diagnosis because of its tissue characterization techniques. Positron emission tomography can be used in certain conditions, especially when CMR is not suitable or when the results are equivocal. Biomarkers of cardiac necrosis, especially cTn (the most specific marker for myocardial injury), are used as a minor criterion for the diagnosis of myocarditis associated with chemotherapy [73].

Patients treated with ICS and diagnosed with myocarditis (associated with chemotherapy) by endomyocardial biopsy on autopsy had elevated levels of cTn (94%), and 46% of these patients developed major cardiac events: cardiovascular death, cardiogenic shock, cardiac arrest, or complete heart block with hemodynamic instability. A cTn level > 1.5 ng/mL was associated with a 4-fold increased risk of major cardiac events (HR: 4.0; 95% CI: 1.5–10.9; *p* = 0.003) [58]. Although elevated levels of cTn are not a specific indicator of cardiotoxicity induced by ICS, they predict a poor prognosis and should be interpreted as an indication of adverse cardiac events [74]. 

A study on 252 patients with lung cancer treated with ICS showed that the incidence of major cardiovascular events was similar in patients treated with ICS and patients treated with other therapies. Nevertheless, ICS-associated cardiotoxicity occurred early during therapy, was dose-independent, and was associated with elevated levels of cTnI [75].

The prompt recognition and risk stratification of myocarditis in patients receiving ICS treatment, especially for those with diabetes, autoimmune diseases, or cardiovascular conditions, can be helpful for reducing mortality and cardiovascular complications. The highest sensitivity for the early diagnosis of myocarditis is obtained by using ECG and hs-cTn; therefore, cTn measurements according to the latest recommendations could be important [71].

The available guidelines of the ESC suggest measurements of cTn before the initiation of treatment and at any moment of the treatment if new cardiovascular symptoms appear during the treatment (e.g., dyspnea, chest pain, palpitations, syncope, or presyncope). In high-risk patients, assessments of cTn should be performed before the second, third, and fourth doses. If the measurements are normal, the measurements can be reduced to alternating doses until the 12th dose, and then every three doses until the completion of treatment [13].

### 2.7. Anti-Vascular Endothelial Growth Factor-Targeted Therapy

Anti-vascular endothelial growth factor (VEGF) therapy includes agents that block the binding of VEGF to receptors, antibodies that arrest the communication through receptors, and tyrosine kinase inhibitors (TKI) that impede the kinase activity on VEGF receptors. These drugs have been associated with hypertension and LV dysfunction, and the possible mechanisms for the development of the latter could be increased ventricular overload, direct toxicity to cardiomyocytes, or microvascular dysfunction [27].

Tyrosine kinase inhibitors (TKI) belong to a class of drugs that target the VEGF receptor, which suppresses the angiogenic response in cancerous tissue. The incidence of asymptomatic LV dysfunction is 30%, and symptomatic heart failure appears in 3–15% of patients receiving TKI treatment [15]. 

Data on the role of cTn in these patients are scarce, and the largest study in this area, which evaluated 90 patients treated with TKI inhibitors, could not find a connection between the elevation of cTn levels and cardiac dysfunction as evaluated by magnetic resonance imaging, echocardiography, and coronary angiograms [76]. At this moment, we do not have an official recommendation for assessing the levels of cTn during treatment with VEGF [27].

## 3. Conclusions

During risk assessment for the cardiotoxicity of different chemotherapy drugs, cTn is an important tool for the early detection of cardiac injury and could predict subsequent changes in the LVEF and the development of HF. The assessment of cTn can be a very useful tool for identifying patients that would benefit from cardiotoxicity prevention treatments, and for monitoring the responses of the patient to treatment. Although much progress has been made so far, and cTn is an important assay in the evaluation of cancer patients, we have little data about the cut-off values of cTn over which patients are certain to develop cardiotoxicity and the levels that suggest a discontinuation of treatment. In this context, an estimation of other confounding factors that could be associated with cTn increases should be assessed and the confirmation of cardiotoxicity should be made by imagistic methods.

An increase in the cTn isolated from other identifiable changes in the cardiac structure and function should not be used as a justifiable reason for chemotherapy cessation. An elevated cTn level should be assessed by the medical team of the patient (oncologist and cardiologist), the risks and benefits should be discussed, and the best decision regarding patient’s treatment and well-being should be taken according to the available information. Elevated cTn levels could lead to increased monitoring frequency, provoke further cardiovascular investigation, or initiate cardioprotective treatment [13]. According to the recommendations of the ESC, patients should be monitored depending on their cardiotoxicity risk, not by their cTn level. If their baseline value of cTn is high, the monitoring frequency should increase and other imagistic methods should be used in order to establish the diagnosis of cardiotoxicity, considering the fact that cTn is not a tool for diagnosis, but a marker of increased risk [8].

## Figures and Tables

**Table 2:** The role of troponin in cardiotoxicity associated with trastuzumab treatment.

Study	Patients	Design	Type of Cancer	Chemotherapy	Type of cTn	Determination of cTn	Outcomes
Cardinale et al., 2010 [59]	251	PR	Breast cancer	TRA	cTnI	Baseline and before and after each trastuzumab cycle	cTnI > 0.08 ng/mL was the strongest independent predictor of cardiotoxicity
Fallah-Rad et al., 2011 [60]	42	PR	Breast cancer	AC, TRA	cTnT	Before AC, before trastuzumab therapy, and 3, 6, 9, and 12 months after the initiation of trastuzumab	cTnT did not show any significant changes over 1 year of follow-up
Ky et al., 2014 [61]	78	PR	Breast cancer	AC, taxanes, TRA	us-cTnI	Baseline and every 3 months (maximum 15 months)	cTnI was associated with the risk of cardiotoxicity
Zardavas et al., 2016 [62]	533	PR	Breast cancer	TRA	us-cTnI hs-cTnT	Baseline; weeks 13, 25, and 52; and months 18, 24, 30, and 36	High-baseline cTnT and cTnI was associated with a 4-fold increased risk of cardiac dysfunction
Yu et al., 2016 [63]	69	Phase 2 sub-study	Breast cancer	Paclitaxel, TRA, pertuzumab	cTnI	Baseline and before every cycle	cTnI (>0.06 ng/mL) was not associated with a decline in LVEF
de Vries Schultink et al., 2017 [64]	206	Secondary analysis of randomized placebo-controlled clinical trial	Breast cancer	AC, TRA	cTnT	Before AC treatment, before starting trastuzumab treatment, and 3, 12, 24, 36, 52, 64, 78, and 92 weeks afterwards	Maximum concentration of cTnT after AC treatment was an important determinant of reduced LVEF
Ponde et al., 2018 [65]	280	Phase III trial sub-study	Breast cancer	Lapatinib, TRA, paclitaxel	cTnT	Baseline and on weeks 2 and 18	No correlation between high cTnT levels and cardiac events
Ben Kridis et al., 2020 [66]	50	PR	Breast cancer	AC, taxanes, TRA	us-cTnI	Baseline and at 3, 6, 9, 12, and 15 months	Levels of us-cTnI at the completion of AC treatment were predictive of the occurrence of cardiotoxicity
Kirkman et al., 2022 [56]	94	Secondary analysis of randomized controlled trial	Breast cancer	TRA	cTnI	Before trastuzumab treatment, post-cycle 4, and post-cycle 17	No significant changes in cTnI were detected

AC—anthracycline, cTn—troponin, hs-cTn—high-sensitivity troponin, LV—left ventricle, LVEF—left ventricular ejection fraction, PR—prospective, TRA—trastuzumab, us-cTnI—ultrasensitive troponin I.

## Data Availability

Not applicable.
